# Supplementary material for: Decoding the Atomic Structure of Ga2Te5 Pulsed Laser Deposition Films for Memory Applications Using Diffraction and First-Principles Simulations
Source: Nanomaterials (Basel). 2023 Jul 23;13(14):2137. doi: 10.3390/nano13142137 (PMC10386151; doi:10.3390/nano13142137)
Supplement: Supplementary file 1 [file nanomaterials-13-02137-s001.zip › nanomaterials-2480081-supplementary.pdf]

nanomaterials

Supplementary Information

Decoding the Atomic Structure of Ga_2Te_5 Pulsed Laser Deposition Films for Memory Applications Using Diffraction and First-Principles Simulations

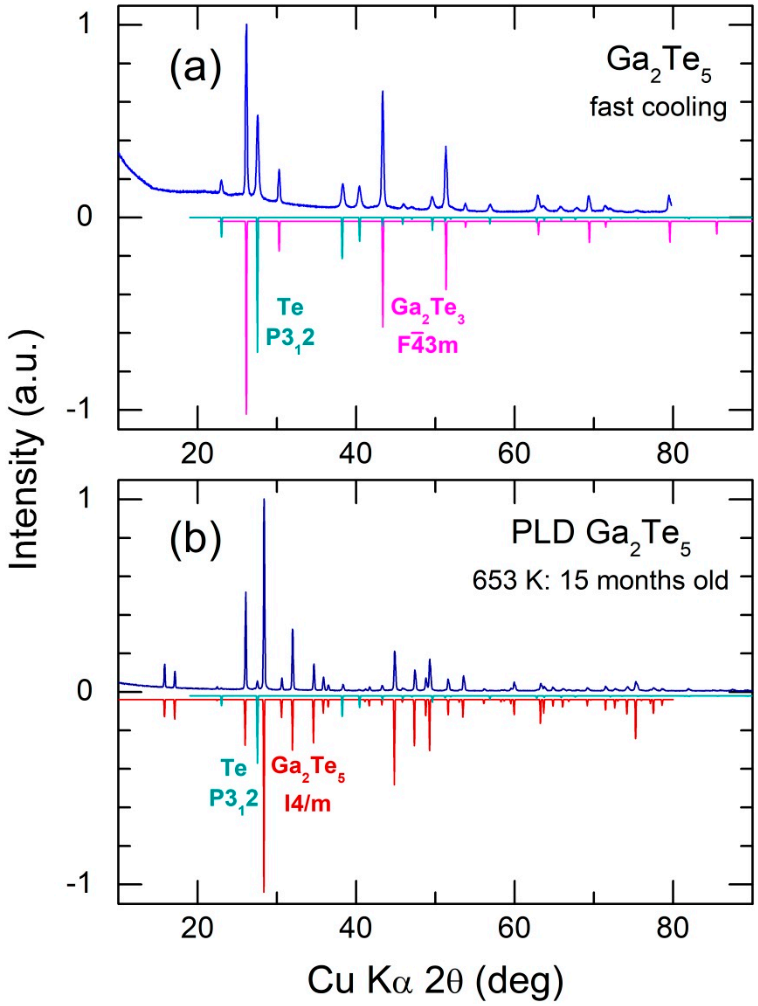

Figure S1. X-ray diffraction patterns of **(a)** rapidly cooled Ga_2Te_5 melt, and **(b)** Ga_2Te_5 PLD film heated to 653 K with a typical DSC rate of 10 K min^{-1}, cooled down to room temperature and stored for 15 months. Rapidly cooled Ga_2Te_5 appears to be a mixture of cubic Ga_2Te_3, space group $F\bar{4}3m$ [1], and trigonal tellurium, $P3_12$ [2]. Annealed 15-month old Ga_2Te_5 PLD film mostly contains tetragonal Ga_2Te_5, $I4/m$ [3], metastable at room temperature [4], and traces of trigonal Te.

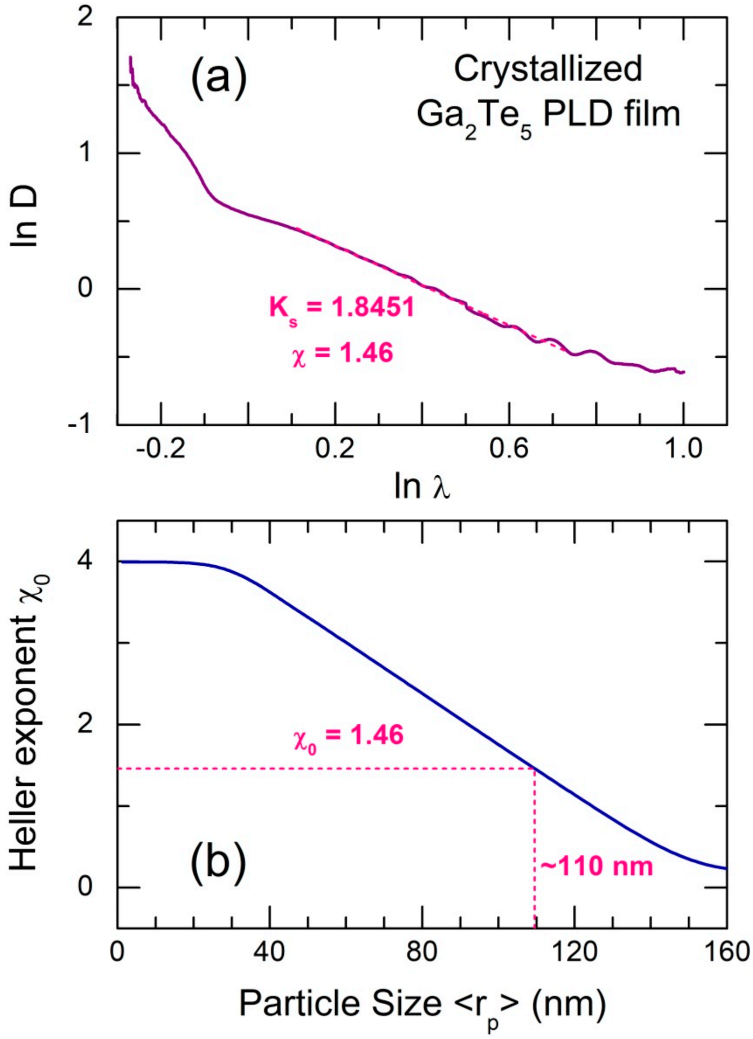

Figure S2. Scattering corrections for crystallized Ga_2Te_5 PLD film: **(a)** optical density D as a function of λ, plotted on a logarithmic scale; the derived scattering coefficient K_S and the wavelength exponent χ are indicated; **(b)** theoretical Heller wavelength exponent χ_0 as a function of the average particle size $\langle r_p \rangle$, yielding $\langle r_p \rangle \approx 110$ nm for the crystallized Ga_2Te_5 PLD film.

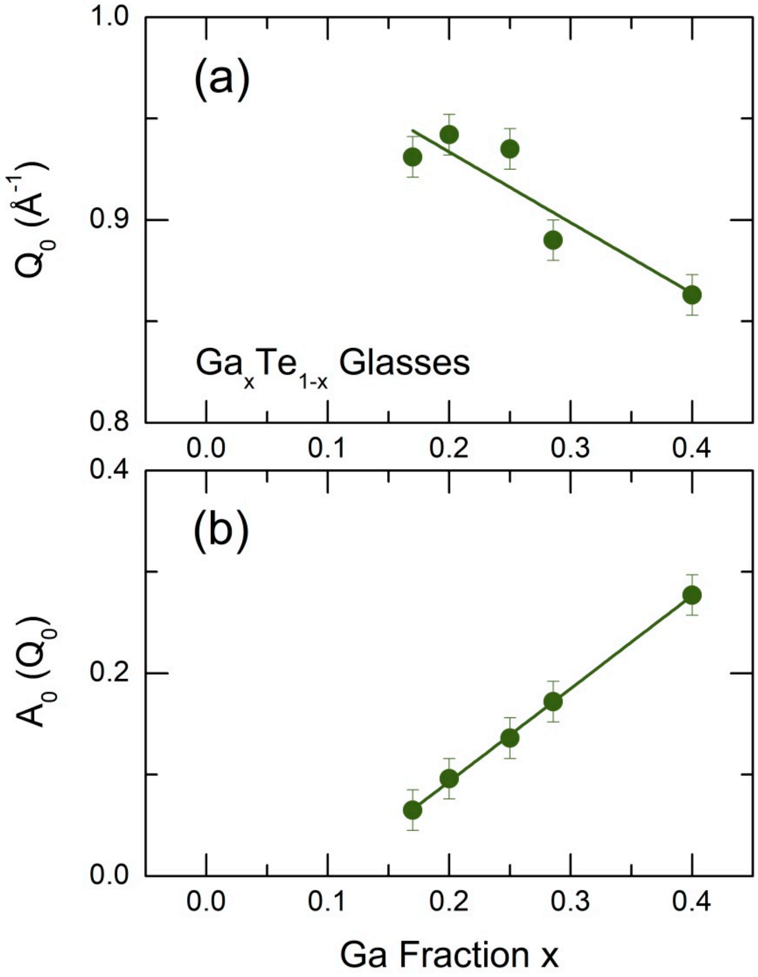

Figure S3. FSDP parameters for glassy Ga_xTe_{1-x} alloys: **(a)** the FSDP position Q_0, and **(b)** the amplitude $A_0(Q_0)$ as a function of x.

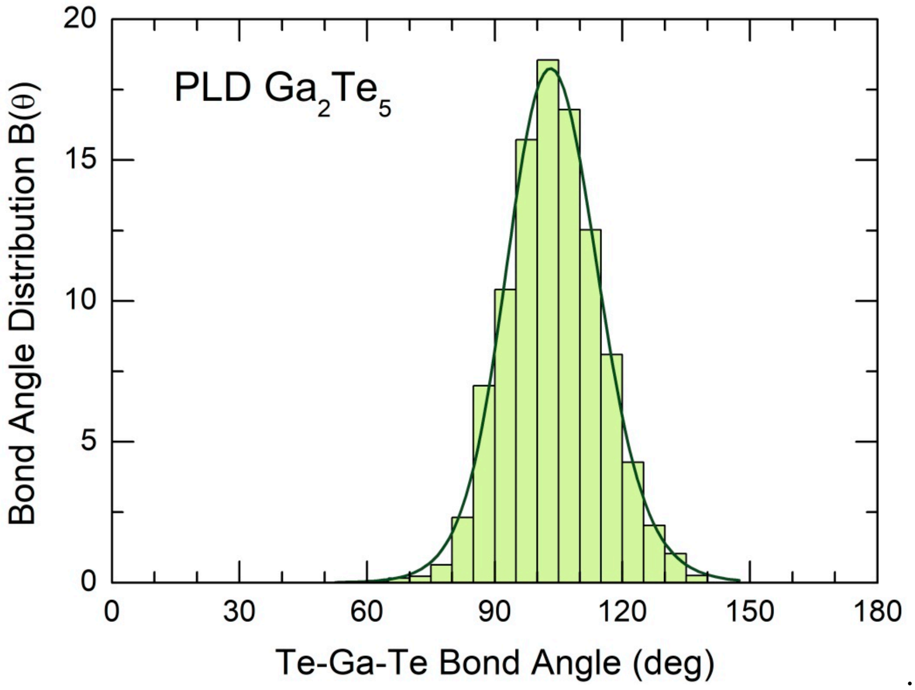

Figure S4. Bond angle distribution $B_{\mathtt{TeGaTe}}(\theta)$ in simulated g-Ga_2Te_5.

References

1. Guymont, M.; Tomas, A.; Guittard, M. The structure of Ga_2Te_3. An X-ray and high-resolution electron microscopy study. *Philos. Mag. A* **1992**, *66*, 133−139.
2. Cherin, P.; Unger, P. Two-dimensional refinement of the crystal structure of tellurium. *Acta Crystallogr.* **1967**, *23*, 670−671.
3. Julien-Pouzol, M.; Jaulmes, S.; Alapini, F. Tellurure de gallium. *Acta Crystallogr. B* **1977**, *33*, 2270–2272.
4. Alapini, F.; Flahaut, J.; Guittard, M.; Jaulmes, S.; Julien-Pouzol, M. Système gallium–tellure. Diagramme de phases, étude structurale de GaTe, Ga_2Te_5 et de Ga_6SnTe_{10}. *J. Solid State Chem.* **1979**, *28*, 309–319.
